# Improvement of production rate on recombinant CHO cells in two-stage culture

**DOI:** 10.1186/1753-6561-7-S6-P50

**Published:** 2013-12-04

**Authors:** Hiroshi Matsuoka, Chie Shimizu, Mihoko Tazawa

**Affiliations:** 1 Dept. Lifesciences, Teikyo University of Science, Tokyo, 120-0045, Japan

## Background

Cultivation temperature is a key environmental parameter that influences cell growth and recombinant protein production. Recombinant CHO (rCHO) cells are usually cultivated at 37 °C. Although lowering culture temperature below 37 °C decrease specific growth rate, in many cases, the specific production rate, *q*, of CHO cells was not enhanced by lowering the culture temperature. Unlike the specific growth rate, effects of low temperature cultivation on specific productivity rate are not so clear [1]. In the present study, we investigated the effect of low temperature cultivation on rCHO cell growth and production rate. We proposed a two-stage culture that the cultivation was carried out at 37 °C and then a culture temperature become lower. We report that the final production concentration by the two-stage culture is higher than that in case of a flat temperature at 37 °C.

## Materials and methods

CRL-10052 was used as the cell line of rCHO, which is the CR1 plasmid was transfected to CHO cells. Target product is the soluble CR1, *s*CR1, which is a soluble form of a human complement receptor type1, could be expressed and secreted by rCHO [2]. Although an original rCHO was an adherent cell, we changed it to be a floating one and used in this experiment. Batch cultivations were carried out in a 1 L-fermentor with a 400 mL working volume at various temperatures. pH and DO were maintained at 7.2 and 40% of air saturation by CO_2 _and O_2_, respectively. Agitation speed was 100 rpm. A serum-free medium on the basis of IMDM with 1% penicillin-streptomycin-neomycin antibiotics mixture was used. An initial cell concentration was 3 × 10^5 ^ml^-1 ^and cultivation was ceased when cell concentration below 1 × 10^5 ^cells mL^-1^. *s*CR1 concentration was determined by using HPLC gel filtration column chromatography (TSK gel G3000SWXL, TOSOH), in which the Tris buffer (pH = 7.4) containing 0.05% CHAPS was used as elution buffer.

## Results

All batch cultivations were carried out until viable cells become equal to zero. Cells grew well at more than 33 °C, however cells didn't grow at 30 °C. Compared to 37 °C-cultivation, lower specific growth rates were observed in the lower temperature cultivations. The specific production rate of *s*CR1, *q_s_*^CR1^, was obtained by the slope of relationship between *s*CR1 concentration and time integrated cell concentration within a linear range. The *q_s_*^CR1 ^at each temperature were the almost same except at 30 ºC.

The final *s*CR1 concentrations at 33 °C was rather higher than those at 37 and 35 °C. The cell concentration in stationary phase, *X*_S_, at 33 °C was lower than those at 37 and 35 °C. Thus the ratio of the final *s*CR1 concentration to *X*_S _at 33 °C was the highest in case of more than 33 °C. The final *s*CR1 concentration to *X*_S _at 30 °C is rather higher than that at 33 °C, however it makes no sense because of the extremely low specific growth rate at 30 °C.

**Figure 1:**
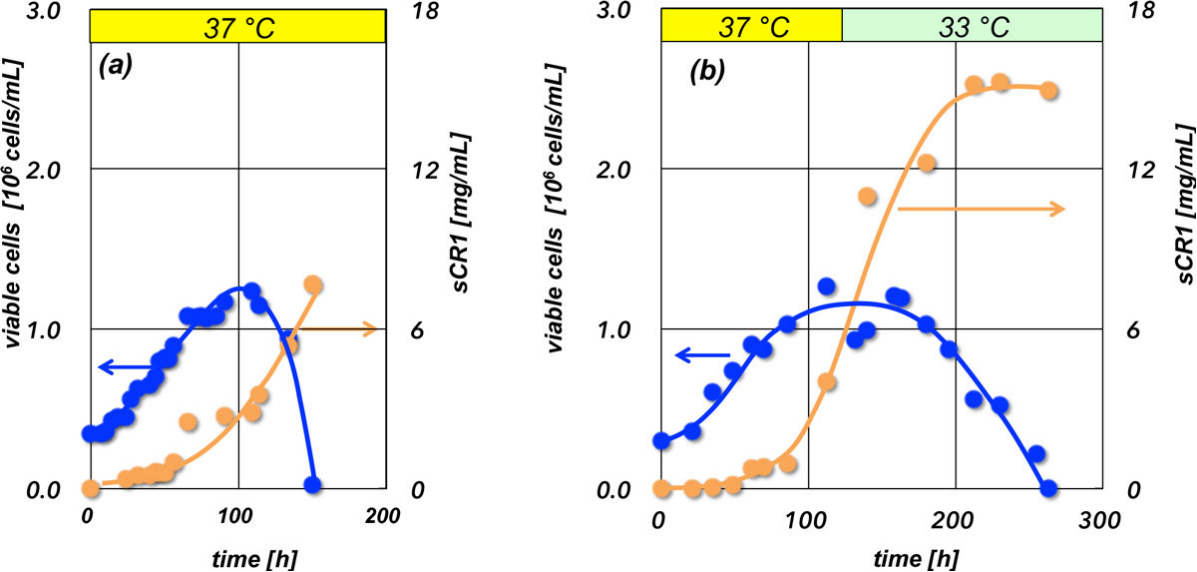
**Time courses of cell-cultivation: (a) 37 °C, (b) two-stage cultivation (37 °C to 33 °C after 120 h)**.

In order to increase the final *s*CR1 concentration, we proposed a two-stage culture that at first cultivation temperature was set to 37 °C and then a culture temperature became lower at late logarithm phase. Thus the final *s*CR1 concentration by using a two-stage culture, in which the temperature was 37 °C initially and changed to 33 °C after 120 h-cultivation, increased by 1.75 and 1.99, compared as a flat temperature culture at 33 °C and 37 °C, respectively (Figure 1, Table 1).

**Table 1:** Comparison of culture parameters at various temperatures

	30 °C	33 °C	35 °C	37 °C	37 °C→33 °C
specific growth rate [h^-1^]	>0.0002	0.0072	0.0107	0.0136	-

*q_s_*^CR1 ^[10^9 ^g cells^-1 ^h^-1^]	0.0304	0.0416	0.0407	0.0446	-

final *s*CR1 [mg/mL] (a)	3.04	8.68	8.11	7.67	15.2

*X*_S _[10^6 ^cells/mL] (b)	0.223	0.788	1.09	1.15	1.20

(a)/(b)	13.6	11.0	7.43	6.68	12.7

## Conclusions

The conclusions are as follows:

1. It was shown that the ratio of the final *s*CR1 concentration to the cell concentration in stationary phase was rather higher at lower temperature than that in 37 °C-cultivation.

2. A two-stage cultivation with temperature change from 37 °C to lower temperature was proposed and it was shown that the final product concentration was considerably improved.
